# The Role of Long-Chained Marine N-3 Polyunsaturated Fatty Acids in Cardiovascular Disease

**DOI:** 10.1155/2012/303456

**Published:** 2012-12-13

**Authors:** Hildegunn Aarsetoey, Heidi Grundt, Ottar Nygaard, Dennis W. T. Nilsen

**Affiliations:** ^1^Department of Medicine, Stavanger University Hospital, 4011 Stavanger, Norway; ^2^Institute of Medicine, University of Bergen, 5020 Bergen, Norway; ^3^Department of Heart Disease, Haukeland University Hospital, 5021 Bergen, Norway; ^4^Department of Cardiology, Stavanger University Hospital, 4011 Stavanger, Norway

## Abstract

This paper reviews the current evidence regarding long-chained marine omega-3 polyunsaturated fatty acids (PUFAs) and cardiovascular disease (CVD), their possible mechanisms of action, and results of clinical trials. Also, primary and secondary prevention trials as studies on antiarrhythmic effects and meta-analyses are summarized. However, the individual bioavailability of n-3 PUFAs along with the highly different study designs and estimations of FAs intake or supplementation dosages in patient populations with different background intake of n-3 PUFAs might be some of the reasons for the inconsistent findings of the studies evaluating the impact of n-3 PUFAs on CVD. The question of an optimum dose of n-3 PUFAs or whether there exists a dose-response relation for n-3 PUFA supplementation is widely discussed. Moreover, the difficulties in interpreting meta-analyses are clearly demonstrated by two recently published meta-analyses (Rizos et al. and Delgado Lista et al.), evaluating the efficacy of n-3 PUFAs on CVD, including 12 common studies, but drawing opposite conclusions. We definitely need more large-scale, randomized clinical trials of long duration, also reporting harmful effects of n-3 PUFAs.

## 1. Introduction

Unsaturated fatty acids (FAs), especially polyunsaturated fatty acids (PUFAs), have since the 1970s been given a lot of attention due to possible health promoting effects. During the first decade of research, pilot studies on Greenland Eskimos demonstrated that a diet rich in long-chained marine PUFAs might reduce the incidence of ischemic heart disease [1]. During the following decades, research has to a large extent focused on the prevention and management of cardiovascular disease (CVD).

 During the same period of time, there has been a large change in our understanding of the atherosclerotic process. From being viewed as inanimate tubes, arteries are now thought of as dynamic tissues, where intimal inflammation plays a crucial role in the pathophysiologic process of atherosclerotic development. A thin fibrous cap is the only structure separating the blood compartment with its coagulation factors from the prothrombotic material in the lipid core. Enhanced inflammation might result in plaque instability. Moreover, the endothelium plays a key role in vascular homeostasis, and endothelial dysfunction seems to be of major importance in the development of a vulnerable plaque. The acute coronary syndrome (ACS) usually results from the erosion or rupture of such a vulnerable atherosclerotic plaque with subsequent coronary artery occlusion as coagulation factors come into contact with tissue factor, the major initiator of the extrinsic coagulation cascade [2]. 

 The complex vascular biology preceding the ACS provides several possible therapeutic targets for PUFAs: reducing atherosclerotic development, stabilizing vulnerable plaques, and limiting the consequences of their disruption. Even though a lot of research has been done trying to elucidate the role of PUFAs in CVD prevention and management, several issues are still under discussion. Clinical studies have provided conflicting results, and the optimal intake of PUFAs is not firmly established. Concerns have been raised about environmental contaminants accumulating in fish, especially methylmercury, polychlorinated biphenyls, and dioxins, giving rise to possible deleterious effects [3] and counteracting the beneficial cardioprotective effects of marine n-3 PUFAs [4]. Especially mercury has been given a lot of attention due to reports of a probable proatherosclerotic effect [5]. N-3 PUFAs might also have a potential to increase oxidative stress, resulting in lipid peroxides [6]. These issues might be related to a dose optimum of n-3 PUFAs, as high doses might exceed an optimum threshold level leading to lack of beneficial effects due to lipid peroxidation or accumulation of other toxic substances. 

 This paper reviews the current evidence regarding long-chained marine omega-3 PUFAs and CVD, their possible mechanisms of action, and results of clinical trials.

### 1.1. PUFAs—Chemistry and Origin

FAs are single-lipid components comprised of a straight hydrocarbon chain terminating with a carboxylic acid group (–COOH) at the polar hydrophilic end and a nonpolar hydrophobic methyl group (–CH_3_) at the other end. The various FAs are named according to their number of carbon atoms and their number and position of carbon-carbon double bonds. FAs with at least two double bonds are designated as polyunsaturated. For PUFAs, the position of the first double bond from the methyl- (n/omega-) end of the molecule has given rise to the terminology n-3 (omega-3) FAs, n-6 (omega-6) FAs, and n-9 (omega-9) FAs [7]. According to the accepted terminology, the number of carbon atoms in a PUFA molecule is designated by the first figure, while the number of double bonds is given by the second figure.

 N-3 and n-6 PUFAs are not synthesized by the human body, as they are essential FAs that need to be ingested [8]. While n-6 PUFAs and *α*-linolenic acid (18 : 3 n-3) are found in vegetable foods, n-3 PUFAs with more than 20 carbon atoms are made by phytoplankton and mainly ingested from fatty fish and marine animals. The two most important marine FAs with respect to human health are thought to be eicosapentaenoic acid (EPA; 20 : 5 n-3) and docosahexaenoic acid (DHA; 22 : 6 n-3). Even though these two FAs to a small extent also can be derived from *α*-linolenic acid by desaturation and elongation in the liver [8], the main body content of EPA and DHA is dependent on the amount ingested. For the present paper we will focus on these long-chained marine n-3 PUFAs.

## 2. Mechanisms of Action of Long-Chained Marine N-3 PUFAs 

The observed health effects of n-3 PUFAs are mainly thought to be mediated by two mechanisms: a change in the properties of the cell membranes and the regulation of gene transcription. As FAs are incorporated into cell membrane phospholipids, the FA composition of this lipid bilayer is reflected by the composition of FAs ingested. Increasing the amounts of n-3 PUFAs in the cell membrane alters its biochemical and physical properties with a subsequent change of membrane fluidity, permeability, and electrophysiological characteristics [9]. Changing membrane properties might further affect the ability of membrane receptors to interact with their ligands or intracellular signalling molecules as well as modulate the effect of membrane bound enzymes [10]. There is also evidence for n-3 PUFAs themselves serving as ligands for nuclear receptors affecting gene expression, nuclear factor *κ*B (NF-*κ*B), and peroxisome proliferator-activated receptor-*γ* (PPAR-*γ*) being two of the potential targets [11–13]. 

Furthermore, 20-carbon PUFAs (arachidonic acid (AA) and EPA) released from cell membrane phospholipids by phospholipase A2 are substrates for the synthesis of eicosanoids, a family of biochemical mediators consisting of prostaglandins (PGs), thromboxanes (TXs), leukotrienes (LTs), and hydroxy fatty acids. Eicosanoids possess important vasoactive regulatory properties, such as regulation of platelet aggregability, endothelial cell motility, cell growth, and chemotaxis [8, 14, 15]. With the increasing incorporation of n-3 PUFAs into cell membranes, the production of eicosanoids is shifted from the 4 series of LTs and the 2 series of PGs and TXs generated from AA to the 5 series of LTs and the 3 series of PGs and TXs derived from EPA, the latter being eicosanoid products leading to a more vasodilatory state, reduced inflammatory responses in the injured vessel wall, and less potent platelet aggregation [8, 14, 15].

A combination of these mechanisms, summarized in Table 1, seems to be responsible for the majority of the proposed antiatherothrombogenic and antiarrhythmic effects of n-3 PUFAs [8, 14, 16, 17] further outlined below. 

### 2.1. Marine N-3 PUFAs and Anti-Atherothrombogenic Effects

In the early 1990s, the extent of atherosclerotic lesions in the coronary arteries and the aortas from Alaskan natives were demonstrated to be significantly lower as compared to non-natives in all age-groups [18]. This was one of the first indications of a possible antiatherosclerotic effect of n-3 PUFAs. In accordance with this finding, a dietary related incorporation of EPA and DHA into advanced human atherosclerotic plaques has been demonstrated [19, 20] along with the development of a more stable plaque morphology, less susceptible to rupture, after n-3 PUFA supplementation [20]. The results from intervention studies on coronary atherosclerosis progression and regression in humans have, however, been highly diverging [21–23].

 The potential effect on atherosclerosis was initially thought to be related to the modulation of proatherosclerotic risk factors. As compared to Danes, Greenland Inuits had lower serum concentrations of total- and low-density lipoprotein cholesterol, triglycerides, and very low-density lipoprotein cholesterol but higher levels of high density lipoprotein cholesterol [1, 24]. Since then, several studies have confirmed that the increasing amounts of n-3 PUFAs are associated with a more favourable profile of lipoproteins [8]. The clear-cut triglyceride-lowering effect of marine n-3 PUFA especially is undisputed [8]. Several mechanisms may contribute to the triglyceride-lowering effect by marine n-3 PUFAs, such as reduced triglyceride synthesis and diminished chylomicron secretion from intestinal cells. Reduced triglyceride formation may partly be due to a reduced pool of fatty acids substrate mediated through suppressed hepatic fatty acid synthesis related to the effect by marine n-3 PUFAs on hepatic gene expression downregulating de novo lipogenesis, increased fatty acid beta-oxidation, and reduced delivery of nonesterified fatty acids to the liver. Besides, marine n-3 PUFAs are poor substrates for enzymes responsible for triglyceride synthesis resulting in reduced and impaired hepatic enzyme activity for triglyceride synthesis and increased hepatic synthesis of phospholipids rather than triglycerides, hereby limiting the secretion from the liver of triglyceride-rich very low-density lipoprotein (VLDL) [8]. 

 Furthermore, ingestion of n-3 PUFAs has been demonstrated to have a blood pressure reducing effect [25], as well as improving glucose metabolism/insulin resistance [26, 27]. The effect of n-3 PUFAs on insulin as a mediator of the metabolic syndrome needs to be clarified, but there is some support for a relationship between the content of n-3 PUFAs in cell membranes and the action of insulin [8]. 

Lately, the role of inflammation and endothelial dysfunction in the pathophysiologic process of atherosclerosis generation and disruption of the vulnerable atherosclerotic plaque has been highlighted [28], opening a new area for potential antiatherosclerotic effects of n-3 PUFAs. The first demonstrated anti-inflammatory effects were related to a shift in the production of eicosanoids by partial replacement of AA by EPA in inflammatory cell membranes, as previously described [8, 14, 15]. During recent years, other anti-inflammatory effects of n-3 PUFAs, independent of the altered eicosanoid production, have been demonstrated. This comprises a reduced production of the proinflammatory cytokines interleukin-1 (IL-1), IL-6, and tumour necrosis factor-*α* (TNF-*α*) from mononuclear cells as well as decreased expression of E-selectin, intercellular adhesion molecule-1 (ICAM-1), and vascular cell adhesion molecule-1 (VCAM-1) on endothelial cells [8], the latter being the molecules essential for the attachment of leucocytes to the endothelium prior to their entrance into the intima. Cellular adhesion molecules are markers of the functional state of the endothelium, and their downregulation by n-3 PUFAs have mainly been demonstrated in studies *ex vivo*. Moreover, beneficial effects on the vasoregulatory secretagogues of the endothelium, such as nitric oxide and prostacycline, have been obtained by n-3 PUFAs [8]. 

The first studies from Bang and Dyerberg on Greenland Eskimos also revealed an antithrombotic effect of high doses of n-3 PUFAs associated with increased bleeding time [29, 30]. However, ingestion of less than 4 grams daily has not been associated with increased risk of bleeding [8, 15, 31]. TXA_2_ is a potent prothrombotic agent, and it is therefore likely that the observed antiplatelet effect of n-3 FAs, at least to some extent, is mediated through a shift in the eicosanoid production. Homocysteine (Hcy) may also exert unfavourable effects on the antithrombotic properties of the endothelium, and in a randomized trial previously presented by our group we observed a reduction of Hcy after treatment with a high dose of n-3 PUFAs as compared to corn oil for 1 year following a myocardial infarction (MI) [32]. The effect of these PUFAs on other haemostatic procoagulant and fibrinolytic factors is, however, divergent [16, 33, 34], and the final impact of n-3 PUFA supplementation on the complex processes of vascular injury, thrombosis, and repair still remains an unsettled issue. 

### 2.2. Marine N-3 PUFAs and Antiarrhythmic Effects

The reduction observed in cardiac death with increased intake of n-3 PUFAs has largely been attributed to a potential antiarrhythmic effect. Studies on cell cultures have revealed that this might be related to a membrane stabilizing effect in cardiac myocytes. Supplementation with n-3 PUFAs has been found to enrich myocyte membranes with EPA and DHA [35–37]. This induces a conformational change of the cell membrane with effects on ion channels and membrane-bound proteins resulting in a slight hyperpolarization of the cell membrane, increasing the depolarizing stimuli necessary to induce an action potential with subsequently reduced automaticity. Furthermore, n-3 PUFAs affect the transition of the voltage-gated sodium channel with a shift toward more negative membrane voltages, promoting recovery from the inactive state and thereby increasing the refractory period. Both effects make the myocardium less excitable, especially in ischemic tissue. In these cells, the negative potential required to reactivate the Na channels might not be physiologically obtainable due to the partial depolarization induced by the dysfunctional state of the Na/K-ATPases. Thus, n-3 PUFAs might be important for opposing the effect of functional re-entry substrates [9]. They are also capable of inhibiting the voltage-dependent inward calcium current during phase 2 of an action potential. In cooperation with possible effects on the Na/Ca exchanger and receptors in the sarcoplasmatic reticulum, this might contribute to less intracellular Ca fluctuations and reduced occurrence of after depolarizations [9]. 

In addition to these direct effects on the generation and duration of the action potential, other less direct mechanisms of actions have been proposed. There is also evidence for antiarrhythmic effects mediated through a reduced production of proarrhythmic eicosanoids, reduced levels of circulating catecholamines [38], and a reduced agonist affinity of beta-receptors [17]. The latter observation might be one of the mechanisms responsible for an improvement in the cardiac sympathetic-vagal balance, revealed clinically as a reduction in the mean heart rate (HR) [39] as well as an increase in HR variability [40]. Both of these parameters have been demonstrated to be related to the risk of malignant arrhythmias and sudden cardiac death (SCD), with increasing HR and decreasing heart rate variability being associated with adverse outcomes [41]. 

Through these mechanisms, n-3 PUFAs seem to be able to interfere with all the proarrhythmogenic mechanisms responsible for the generation of ventricular arrhythmias. 

In agreement with these proposed mechanisms, animal experiments have demonstrated a beneficial effect of EPA and DHA on the development of ischemia-induced ventricular arrhythmias [42], whereas results from human studies are more divergent. For ventricular arrhythmias generated by other mechanisms, such as myocardial scaring and heart failure, the same protective effect of n-3 PUFAs might not be present, as suggested by the highly discrepant results of n-3 PUFA supplementation in patients with an implantable cardioverter defibrillator (ICD) [43–47].

## 3. The Omega-3 Index

 The omega-3 index is defined as the content of EPA and DHA in the cell membrane of red blood cells (RBCs), expressed as a weight percentage of total FAs. The omega-3 index correlates highly with the EPA + DHA content in serum, plasma, and whole blood [48, 49], but as opposed to these findings, RBC EPA + DHA is better correlated to long-term FA intake as evaluated by a food frequency questionnaire (FFQ) and is a more suitable biomarker for the nutritional status of an individual [49]. The half-life of EPA + DHA in RBCs is 4–6 times longer than in serum [50], with concentrations returning to baseline 16 weeks after supplementation [51]. 

In addition, the omega-3 index has been found to be highly correlated with cardiac EPA + DHA levels and responds to supplementation in a way very similar to that of myocardial tissue [35, 36]. With RBCs readily available and easy to analyze, this gives us the opportunity to apply the omega-3 index as a surrogate for cardiac omega-3 status in clinical practice. 

## 4. N-3 PUFAs and Relation to Cardiovascular Disease

The individual bioavailability of n-3 PUFAs along with highly different designs, estimations of FA intake, or supplementation dosages might be some of the reasons for the inconsistent findings of studies evaluating the impact of n-3 PUFAs on CVD. A summary of the existing knowledge will be given below. 

### 4.1. Primary and Secondary Prevention Trials

The first evidence of a possible health effect in humans with a diet rich in n-3 PUFAs came from the pioneer studies on Greenland Eskimos in the 1970s [1], reporting lower coronary mortality in this population than in Danish control subjects. Since then, a huge amount of the literature has been published, further investigating the effects of these marine-derived PUFAs on CVD. Two of the most cited studies are the Diet and Reinfarction Trial (DART) investigating the effect on recurrent cardiac events of dietary advice after a recent MI [52] and the GISSI-Prevenzione trial in which marine-derived n-3 PUFA supplementation was given for the prevention of death, nonfatal MI, and stroke in patients who had survived an MI during the preceding 3 months [53]. In the DART study, 2 years of followup revealed a 29% reduction in total mortality (*P* < 0.05) among patients advised to eat fatty fish, mainly due to a reduction in deaths from coronary artery disease (CAD). Comparable to this finding, supplementation with 1 g/day of n-3 PUFAs (*≈*850 mg/day of EPA and DHA ethyl esters in a ratio of 1 : 2) for 3.5 years in the open label GISSI-Prevenzione study reduced the composite endpoint of death, nonfatal MI, and stroke by 15% (*P* = 0.023). In subgroup analyses, the reduction in relative risk was even greater for cardiovascular death and SCD which was reduced by 30% (*P* = 0.024) and 45% (*P* = 0.01), respectively. 

It has been argued that the GISSI-Prevenzione trial was not designed to evaluate SCD, and that it had insufficient statistical power. The results of these subgroup analyses should, therefore, be interpreted with caution. Furthermore, there has clearly been an evolvement in the general treatment of CAD from the time of performance of the GISSI trial until today, and it has been argued that supplementation with similar doses of n-3 PUFAs in addition to current guideline-adjusted therapy might not have the same benefit. The participants of the GISSI trial were included in the early 1990s, at a time when medical prescriptions and the use of early coronary revascularization were quite different from today's management. To investigate this question, Rauch et al. conducted the Omega trial, a randomized, placebo-controlled, and double-blind trial evaluating the effects of 1-year treatment with 1 g/day of n-3 PUFAs (380 mg DHA + 460 mg EPA) following an MI [54]. Treatment was initiated 3–14 days after MI in 3851 patients. As compared to the GISSI trial in which 5% of the patients received coronary revascularization at baseline, 93.8% of the participants of the Omega trial underwent acute percutaneous coronary intervention (PCI). The Omega trial revealed no effect on the rate of SCD (*P* = 0.84), total death (*P* = 0.18), nonfatal reinfarction, stroke (*P* = 0.10), or revascularization procedures in survivors (*P* = 0.34) after 1 year. The Omega trial might, however, have been underpowered in an era of more aggressive risk factor management. 

No effect of n-3 PUFA supplementation could be demonstrated on the risk of major cardiovascular events (fatal and nonfatal CVD and cardiac intervention with PCI or coronary artery bypass grafting (CABG)) in the Alpha Omega Trial [55] in which 4837 patients receiving state-of-the-art medical therapy after MI were randomized to one of four margarines for 40 months; a margarine supplemented with a combination of EPA and DHA, a margarine supplemented with alpha-linolenic acid (ALA), a margarine supplemented with EPA/DHA and ALA, or a placebo margarine. The margarine compounds resulted in an uptake of 226 mg of EPA combined with 150 mg of DHA, 1.9 g of ALA, or both, respectively. As compared to placebo, neither EPA/DHA nor ALA reduced the occurrence of the primary endpoint (HR with EPA/DHA 1.01 (95% CI 0.87–1.17; *P* = 0.93); HR with ALA 0.91 (95% CI 0.78–1.05, *P* = 0.20)). However, the dose of marine n-3 PUFA used in this study was less than half of the dose ingested in the GISSI trial [53].

As opposed to these studies, the GISSI-HF trial provided evidence for a small, but beneficial, advantage of n-3 PUFA supplementation on risk of total mortality (HR 0.91, 95.5% CI 0.833–0.998, *P* = 0.041) and combined risk of total mortality or admission to hospital for cardiovascular reasons (HR 0.92, 99% CI 0.849–0.999, *P* = 0.009) in patients with heart failure of any cause and irrespective of left ventricular ejection fraction. These patients were given standard care according to guidelines in the early 2000s [56]. In absolute terms, 56 patients needed to be treated for a median duration of 3.9 years to avoid one death or 44 to avoid one combined event of death or admission to hospital for cardiovascular reasons. In this randomized, double-blind, and placebo-controlled trial, patients were given 1 g n-3 PUFAs daily (*n* = 3494) or placebo (*n* = 3481) and followed for a median of 3.9 years. Most interestingly, the same study has demonstrated that n-3 PUFAs can provide a small but significant improvement of left ventricular function in patients with symptomatic heart failure [57]. Left ventricular ejection fraction increased with n-3 PUFAs by 8.1%, 11.1%, and 11.5% after 1, 2, and 3 years, respectively. In the placebo group, the corresponding changes from baseline were 6.3%, 8.2%, and 9.9% (*P* = 0.005). This finding has recently been verified in two smaller groups of patients with nonischemic dilated cardiomyopathy receiving 1–4 g/day of n-3 PUFAs versus placebo for 3–12 months [58, 59]. In one of these studies, administrating supplements for only 3 months, a dose-dependent increase in left ventricular function was observed [58]. 

As opposed to the DART and GISSI trials, two recent studies from Japan observed the reduction in risk of CAD to be primarily related to nonfatal coronary events. The Japan Public Health Centre-based (JPHC) study included a total of 41 578 Japanese men and women aged 40–59 years initially free of CVD [60]. After 10 years of followup, there was a 57% reduction in risk of nonfatal cardiac events for the highest as compared to the lowest quintile of fish intake [HR = 0.43 (0.23–0.81)], while no favourable effect was observed on fatal events or SCD. In the Japan EPA Lipid Intervention Study (JELIS), supplementation with 1800 mg EPA in combination with a statin was given to 18 645 hypercholesterolemic subjects (total cholesterol ≥ 6.5 mmol/L) for a median time of 4.6 years [61]. Twenty percent of these individuals presented with established CAD. As compared to statin treatment alone, there was a 19% relative reduction in the primary outcome measure including SCD, fatal, and nonfatal MI and other nonfatal events such as unstable angina pectoris, angioplasty, stenting, and coronary artery bypass grafting (*P* = 0.011). Subgroup analyses demonstrated, however, beneficial effects mainly in the setting of secondary prevention with a significant reduction in risk of unstable angina of 28% (*P* = 0.019). 

The intake of n-3 PUFAs in these Japanese studies was quite high, with participants of the upper quintile of fish intake in the JPHC study having fish servings at least 8 times per week and the population as a whole having a mean intake of 900 mg n-3 PUFAs/day. In a review of prospective cohort studies and randomized controlled trials, Mozaffarian and Rimm [62] demonstrated evidence for a maximal risk reduction of death from CAD with servings amounting to about 250 mg n-3 PUFAs/day. We recently observed a similar though nonsignificant threshold effect of dietary n-3 PUFAs on risk of coronary events among patients undergoing coronary angiography for suspected coronary artery disease [63]. Thus, for populations already consuming 250 mg/day of EPA + DHA, no further risk reduction for cardiac death seems to be achieved. This threshold-related effect may explain the lack of mortality benefit observed in the JPHC and JELIS studies. Their background fish intake was associated with very low coronary heart disease death rates (87% lower than in comparable Western populations), and additional n-3 PUFA intake yielded little further reduction in the death rate, as most of the population was already above the threshold for maximum mortality benefit. In the JPHC study, only subjects with a mean daily intake of 2.4 g n-3 PUFAs had any reduced risk of nonfatal events, indicating that even greater dosages might be needed to reduce the risk of nonlethal CAD events. 

The choice of dose (1 g of n-3 fatty acids containing 465 mg EPA and 375 mg DHA per day) and the relatively high background intake of n-3 PUFAs reported in the ORIGIN trial (the Outcome Reduction with an Initial Glargine Intervention) [64] might explain the lack of beneficial effects of n-3 PUFAs on cardiovascular mortality and morbidity in this study. This international, multicenter, randomized, and open-label trial with a 2 × 2 factorial design evaluated the protective effects of n-3 PUFAs in a daily dose of 1 g versus corn oil, and Insulin Glargine (Lantus) versus standard care, on cardiovascular mortality and morbidity during 6 years of followup in 12 536 high-risk subjects with impaired fasting glucose, impaired glucose tolerance, or early type 2 diabetes. The estimated median dietary intake of n-3 PUFAs of 210 mg/day might have muted the potential effects of n-3 treatment, assuming that maximal risk reduction is obtained by consuming 250 mg n-3 PUFAs/day, as suggested by Mozaffarian and Rimm [62]. 

Several other trials have confirmed an inverse association between intake of n-3 PUFAs and risk of CAD [3, 65–70], especially fatal cardiac events [65, 67–70]. This finding has been evident both in the setting of primary [3, 65, 66, 68–70] and secondary prevention [52, 53, 67]. Only a few epidemiological studies and randomized controlled trials have presented results indicative of a lack of effect [54, 55, 71–74] or a direct harmful effect of a high intake of n-3 PUFAs [75, 76]. The lack of effect on CAD from a relatively high intake of n-3 PUFAs in Western coastal populations has been suggested to be due to a concomitant high intake of saturated FAs and monounsaturated FAs [77]. The Western coastal populations differ not only from the Japanese with respect to n-3 PUFA levels; they also ingest more of the apparently unhealthy FAs with a possible attenuation of the health effects of n-3 PUFAs. In this setting we cannot rule out the harmful effects of environmental contaminants of fish and increases of oxidative stress, as previously discussed. The major clinical trial data for primary and secondary prevention of CVD are summarized in Table 2(a).

### 4.2. Studies on Antiarrhythmic Effects

Time-course analyses of the GISSI-Prevenzione study with a reduction in SCD already after 4 months of supplementation [83] have given rise to the hypothesis of a predominant antiarrhythmic effect of n-3 PUFAs. This hypothesis is supported by a case control study including 334 patients with primary cardiac arrest performed by Siscovick et al. [84] who demonstrated that an intake of 5.5 g of n-3 PUFAs per month (equivalent to one fatty fish meal per week) as compared to no intake was associated with a 50% reduction in the risk of cardiac arrest (95% CI 0.4–0.8). All cases and controls were free of prior clinical heart disease. In the same study, there was an inverse relationship between blood measurements of EPA + DHA and risk of cardiac arrest. The same has been evident for SCD in the Physicians' Health Study [85] and for fatal ischemic heart disease in the Cardiovascular Health Study [86]. In the latter study, patients over the age of 65 had a 77% lower risk of assumed arrhythmic death for each standard deviation increase in plasma phospholipid DHA + EPA. None of these studies can, however, document an antiarrhythmic mechanism of protection against SCD, as the electrical activity of the myocardium at the moment of cardiac arrest was not systematically registered.

The first evidence of an antiarrhythmic potential of n-3 PUFAs came from experimental work in animal models [87–90], and in a systematic review and meta-analysis of the impact of n-3 PUFAs on selected arrhythmia outcomes in these animal models, Matthan et al. [90] conclude that there is a beneficial effect of EPA and DHA on ischemia-induced ventricular fibrillation (VF) and ventricular fibrillation (VT) across all species. For ventricular arrhythmias induced by reperfusion, the results were inconsistent, and none of the animal models evaluated other arrhythmogenic mechanisms, such as scar-related malignant arrhythmias. 

In attempts to determine whether n-3 PUFAs could have the same antiarrhythmic effects in humans, several studies have been performed in ICD patients with a high risk of recurrent ventricular arrhythmias. Schrepf et al. [45] were able to abort the inducibility of VT in 5 out of 7 ICD patients undergoing electrophysiological testing by intravenously infusing 3.8 g of n-3 PUFAs. The same finding was recently published by Madsen et al. [81] testing eight ICD patients undergoing a randomized, placebo-controlled, crossover study with electrophysiological testing performed both after infusion of 3.9 g of n-3 PUFAs and placebo. Of the 5 patients who were inducible after placebo, 2 were no longer inducible after n-3 PUFAs infusion, and another 2 required stronger stimulation to induce VT. Comparable to these findings, Christensen et al. [46] have also demonstrated that ICD patients with a low content of n-3 PUFAs in serum have a higher incidence of ventricular arrhythmias as compared to patients with high serum levels (*P* < 0.05). 

Results have been less consistent in studies of orally administered supplementation of n-3 PUFAs. Leaf et al. [47] could only demonstrate a trend toward prolonged time to the first ICD event (VF or VT) for patients receiving 4 g/day of fish oil supplements (total dose of EPA and DHA of 2.6 g/day) as compared to olive oil supplements for 12 months, while Metcalf et al. [44] found that a daily dose of 3 g encapsulated fish oil for approximately 6 weeks resulted in noninducible or less inducible VT in a group of patients with ischemic cardiomyopathy. Brouwer and colleagues [78] found no strong evidence for a protective effect of a daily dose of 0.9 g n-3 PUFAs for 1 year as compared to that of sunflower oil in 546 ICD patients. A similar dose (1 g/day of n-3 PUFAs) did, however, result in a trend toward protection in a substudy of the GISSI-HF trial when given to 566 patients for a median followup duration of 928 days [79]. There was a nonsignificant 20% reduction in appropriate ICD managed VT/VF events in the n-3 PUFA group as compared to placebo. Interestingly, this did not result in any mortality benefit. There was actually a minimal excess in total mortality observed in the group treated with n-3 PUFAs (26.6% versus 24.3%). This is in some contrast to the results of the main study where the greatest proportion of the absolute risk reduction of total mortality by n-3 PUFA supplementation was attributable to a reduction in presumed arrhythmic deaths [56]. Moreover, in the study by Raitt et al. [80], recurrent episodes of VT or VF during 2 years of followup occurred more frequently and with reduced time to event in patients receiving 1.3 g/day of EPA/DHA as compared to olive oil. 

As previously mentioned, the protective effect of n-3 PUFAs might be strongest for ischemia-induced ventricular arrhythmias. The implantation of an ICD is more often performed in the setting of VF or VT without a concomitant MI or any reversible cause or in the case of high risk of SCD, such as excessive heart failure or demonstration of inducible VF/VT at electrophysiological examination. Even though ischemia might contribute to recurrent arrhythmic events, these patients may also have other arrhythmic substrates which to a lesser degree may be affected by n-3 PUFAs. The mixture of both VF and VT as a primary endpoint in most of the ICD studies also complicates the comparisons. The study by Christensen et al. [46] demonstrating a significantly lower frequency of ICD events for patients with the highest serum content of n-3 PUFAs reported only a reduction in the presence of VF. Even though a reduction in inducible VT has been achieved in both animals and humans after infusion of n-3 PUFAs as well as after high-dose supplementation [44], it is not known whether these marine PUFAs in normal dietary or supplementary ingested doses have the same potential to reduce the occurrence of spontaneous VT as compared to VF. It is, however, interesting to note that those studies reporting no beneficial effect of oral supplementation seem to have the highest proportion of patients with only VT prior to inclusion [78, 80]. Furthermore, there is a high diversity between the ICD studies in supplementation doses and the length of the intervention, making an overall conclusion even harder to achieve. 

In agreement with a possible antiarrhythmic effect in the setting of ischemia-induced ventricular arrhythmias, we have recently presented evidence for a reduced risk of VF during the acute ischemic phase of an MI with high levels of cellular EPA and DHA [91]. After adjustment for other potential predictors of risk of VF, our case-control study including 10 patients with VF during the initial 6 hours of symptom onset suggested a 48% reduction in risk of this life-threatening arrhythmia with a 1% increase of the omega-3 index. We have later been able to reproduce the same finding in another SCD population comprising 12 case patients with a first-time MI [82]. The main strength of both of these studies is the documentation of VF at the time of cardiac arrest, lending support to the hypothesis of an antiarrhythmic effect of n-3 PUFAs. Interestingly, results of the electrophysiological studies performed both in animals [88] and humans [45, 81], demonstrating reduced inducibility or termination of ventricular arrhythmias immediately after IV infusion of n-3 PUFAs, also suggest that incorporation of marine PUFAs into cell membranes might not be necessary for their antiarrhythmic effect. The major clinical trial data on antiarrhythmic effects are given in Table 2(b).

### 4.3. Meta-Analyses

A meta-analysis by Hooper et al. published in Britical Medical Journal (BMJ) in 2006 was given a lot of attention in media due to the conclusion that n-3 PUFAs given for at least 6 months had no clear effect on total mortality or combined CVD events [92]. This analysis was based on 41 cohort studies and 48 randomized intervention trials including both patients with and without established CVD but has later been criticized for several methodological problems. A recently published meta-analysis by Filion et al. [93] comprising 29 randomized controlled trials (RCTs) including 35 144 high-risk CVD patients could neither demonstrate a statistically significant decrease in total mortality (RR 0.88, 95% CI 0.64–1.03) nor an effect on restenosis prevention (RR 0.89, 95% CI 0.72–1.06) by n-3 PUFAs. 

The results of these reviews differ, however, from several other meta-analyses evaluating observational studies and RCTs [94–99]. They all demonstrate a reduced risk of cardiac death with increasing intake of marine PUFAs. The review by He et al. [95] could actually demonstrate a 7% lower risk of coronary heart disease mortality for each 20 g/day increase in fish intake. The results are less consistent when evaluating the effect of n-3 PUFAs on nonfatal CVD events. Yzebe and Lievre [96], including 10 RCTs comprising 14 727 patients in their analysis, found no significant effects on nonfatal MI, nonfatal stroke, or the presence of angina pectoris. The same lack of influence by n-3 PUFAs on the incidence of nonfatal MI was observed by Bucher et al. [99]. This is opposed to the outcome from the review of 11 RCTs including 39 044 patients performed by Marik and Varon [97] where dietary supplementation with n-3 PUFAs significantly reduced the risk of nonfatal CVD events (OR 0.92, *P* = 0.02). The latter finding might, however, be due to the fact that 48% of the patients included in that meta-analysis belonged to the JELIS study [61]. Furthermore, the heterogeneity of the effect of n-3 PUFA intake on CVD outcome might be related to the impact of varying doses and time of followup. 

In a meta-analysis by Kwak et al. [100] involving 20 485 patients with a history of cardiovascular disease from 14 randomized, double-blind, and placebo-controlled studies, intervention with EPA and DHA showed insufficient evidence of a secondary preventive effect of n-3 PUFA supplements against overall cardiovascular events. JELIS [61] and GISSI 4 [53] were excluded from this analyses, as these were open studies.

The reduced risk of cardiac death associated with the intake of n-3 PUFAs has largely been attributed to the prevention of SCD. Two meta-analyses have specifically evaluated this outcome. Both Marik and Varon [97] and Chen et al. [98] found a reduced risk of SCD to be associated with the intake of n-3 PUFAs. In the study by Chen et al., this effect was, however, limited to CVD patients without guideline-adjusted therapy (RR 0.64, 95% CI 0.51–0.80). 

There are only a couple of meta-analyses available regarding the effect of n-3 PUFAs in ICD patients [43, 101], both of which include the previously described studies by Leaf et al. [47], Brouwer et al. [78], and Raitt et al. [80]. None of these studies support a protective effect of n-3 PUFAs from fish oil on cardiac arrhythmia in patients with an ICD.

However, the difficulties in interpreting meta-analyses are clearly demonstrated by the recently published two meta-analyses by Rizos et al. [102] and Delgado-Lista et al. [103] evaluating the efficacy of omega-3 PUFAs on CVD, including 12 of the same studies, but drawing opposite conclusions. Both include randomized controlled trials in primary and secondary prevention, but the endpoints differ. Rizos et al. [102], including 20 studies, conclude that omega-3 PUFA supplementation was not associated with a lower risk of all-cause mortality, cardiac death, sudden death, myocardial infarction, or stroke, based on relative and absolute measures of association. On the contrary, Delgado-Lista et al. [103], including 21 studies, conclude in favour of omega-3 fatty acids, stating that marine omega-3 fatty acids are effective in preventing cardiovascular events of any kind (composite endpoint of stroke, coronary events, myocardial infarction or angina pectoris, peripheral limb disease events, or death from cardiovascular causes), cardiac death, and coronary events, especially in persons with high cardiovascular risk. 

### 4.4. Studies on Triglycerides

The triglyceride-lowering effect of marine n-3 PUFAs is well recognized and has been demonstrated to be linearly dose-dependent across a wide range of consumption. As early as in 1990, Schmidt et al. performed their dose-response studies on the effect of marine n-3 PUFAs on triglyceride levels, showing that 6 weeks of supplementation with 1.3, 4, and 9 grams of n-3 PUFAs daily to healthy normolipidemic men resulted in a reduction in plasma triglycerides by 9%, 25%, and 33%, respectively, in response to increasing doses of n-3 PUFAs [33]

The minimal effective dose of marine n-3 PUFAs has been demonstrated to be about 1 gram daily, also in accordance with observations in the GISSI study, where 1 g marine n-3 PUFAs for 6 months resulted in a small, but significant, triglyceride reduction [53]. 

Even greater dose-dependent reductions in plasma levels of triglycerides of 40%–50% have been observed among individuals with hypertriglyceridemia. The American Heart Association (AHA) [104] recommendations state that EPA-DHA supplements may be useful in patients with severe hypertriglyceridemia (>500 mg of triglycerides per deciliter (5.6 mmol per liter)) in doses of 2 to 4 g of EPA-DHA per day to lower triglyceride levels by 20% to 40% in people in whom diet and lifestyle measures have not led to appropriate concentrations of triglycerides. This is also in agreement with the US National Cholesterol Education Program (NCEP) [105]. However, a meta-analysis studying the triglyceride-lowering effect of marine n-3 PUFAs in a daily dose of 4 grams administered to subjects with moderate hypertriglyceridemia (triglycerides 150–500 mg/dL) concludes that marine n-3 PUFAs are effective in reducing triglycerides by approximately 30% [106]. Additional lipid disturbances and CVD risk factors should be considered before therapeutic decisions are made. Although clinical studies have reported considerable triglyceride-lowering effects by marine n-3 PUFA supplementation, no data for clinical endpoints are available to lend support to this recommendation. Further investigation is needed to explore this area.

## 5. The Omega-3 Index—A New Risk Factor for CAD?

 The omega-3 index has during the recent decade been proposed as a new risk factor for CVD, especially for fatal cardiac events [48, 107, 108]. This index is an independent measurement of the amount of EPA and DHA available in the body, highly reflecting the FA composition of the myocardium [35–37]. In case-control studies the omega-3 index has been demonstrated to be an independent risk marker for SCD [84] and for the development of an ACS [109, 110]. 

We could, however, not demonstrate any prognostic utility of the omega-3 index in the setting of secondary prevention in 460 ACS patients from the “Risk Markers in the Acute Coronary Syndrome study” (RACS) [111] with respect to future cardiac events or risk of death after adjustment for traditional risk factors and established risk markers. We have, however, in two different populations of first-time MI-patients demonstrated low levels of the omega-3 index to be independently associated with increased risk of cardiac arrest/VF during the acute ischemic phase of an MI [82, 91]. 

The omega-3 index appears to fulfill many of the criteria required for a risk marker/risk factor, especially for SCD [48, 107, 108]. It has been estimated that the highest risk of cardiac death is associated with an omega-3 index below 4%, with a level of 8% offering the greatest degree of cardioprotection [48, 108]. These estimates correspond well with the actual measurements in our cardiac arrest patients but could not be confirmed as cut-points to classify patients at low, intermediate, or high risk in our prognostic study. Further studies are needed to elucidate the final role of the omega-3 index in a clinical setting. 

## 6. Conclusions and Further Perspectives

 Despite a significant amount of research since the pioneering work of Bang and Dyerberg in the 1970s attempting to elucidate the possible health effects of n-3 PUFAs, there are still several areas of uncertainty. The existing literature is, however, mainly supportive of a cardioprotective effect of marine fish oils, even though there is some disagreement as to whether this effect is mediated through a reduced risk of fatal or nonfatal cardiac disease. This might, however, be a question of an optimum dose or whether there exists a dose-response relation for n-3 PUFA supplementation. A final conclusion is difficult to reach, as studies differ in design and are performed in highly different patient populations with different background intake of fish and reflect the administration of a wide range of supplement doses of n-3 PUFAs for varying periods of time. We definitely need more high quality, large-scale, randomized, and controlled clinical trials of long-term duration also reporting possible harmful effects. Furthermore, studies on out-of-hospital cardiac arrest will hopefully answer some more questions related to the association between n-3 PUFAs and risk of SCD. Moreover, to diminish difficulties in interpreting meta-analyses, future meta-analyses should strive to include trials with more homogenous designs and populations with respect to cardiovascular risk profile, using similar doses of marine n-3 PUFAs, with precisely defined endpoints. 

We are still awaiting results of three large-scaled trials which might contribute to a further understanding of the appropriate role of n-3 PUFAs in the primary prevention of cardiovascular events in both high- and low-risk participants. In the large ASCEND (A Study of Cardiovascular Events in Diabetes) trial, 15 480 diabetic patients (type I and II) without CVD at the time of inclusion were randomized with a 2 × 2 factorial design to receive low-dose aspirin (100 mg), n-3 PUFA 1 g/day, both regimens or placebo in a 2 × 2. Followup is scheduled to continue until 2017. No publication is yet available. The Rischio and Prevenzione study has between 2004 and 2007 randomized 12 513 participants without a previous MI to a daily dose of 1 g of n-3 PUFAs or placebo (olive oil) for 5 years to evaluate the influence of n-3 PUFAs on death or hospitalization for cardiovascular events. Finally, the third large study, the Vitamin D and Omega-3 Trial (VITAL), is currently recruiting 20 000 participants in the US without a history of CVD and cancer, for supplementation with Vitamin D and/or 1 g n-3 PUFAs, to assess the preventive effects of n-3 PUFAs on these conditions. 

The official recommendations for the use of marine n-3 fatty acids in CVD prevention by major official organizations such as The American Heart Association (AHA), the European Society of Cardiology (ESC), the International Society for the Study of Fatty Acids and Lipids (ISSFAL), and the World Health Organization (WHO) are given in Table 3. However, based on the existing knowledge, the general recommendation of fish or fish oil supplements in patients at risk of, or with established CVD, has not been substantiated, but high doses of n-3 PUFAs appear to be safe in combination with current recommendations regarding medical treatment of CAD patients, especially when combined with aspirin and clopidogrel [112]. 

## Figures and Tables

**Table 1:** Mechanisms and biochemical effects of marine PUFA.

Anti-inflammatory effects	
(i) Competition with AA for Cox/lipooxygenase sites	
(ii) Increase of anti-inflammatory eicosanoids	
(iii) Reduction of TNF*α*, IL-1, IL-6	
(iv) Reduction of nuclear factor *κ*B (NF- *κ*B) activation	

Vascular effects	
(i) Increased vagal tone	
(ii) Improved endothelial function	
(iii) Increase of NO	
(iv) Reduction of Hcy, VCAM-1, ELAM-1, and ICAM-1	
(v) Reduction of ET-1	

Antithrombotic effects	
(i) Reduced platelet aggregation via reduction in TXA2	
(ii) Increased bleeding time (high doses)	

Triglyceride-lowering effect	

Antiarrhythmic effects	
(i) Increased membrane stabilization, reduced automaticity, and increased refractory period	
(ii) Increased EPA : AA ratio in plasma membrane of cardiac myocytes	
(iii) Reduced production of proarrhythmic eicosanoids	
(iv) Reduced agonist affinity of beta-receptors → reduced heart rate (HR), increased HR variability	
(v) Inhibition of the L-type calcium current	
(vi) Inhibition of fast voltage-dependent Na^+^ channels	

**(a):** 

Study	Dose of FAs	Control	*N*	Followup	Prior MI/CAD	Mortality			Nonfatal MI	All CVDevents	Stroke
						All-cause	Cardiac	SCD			
RCTs, blinded											

Omega [54]	1 g/d n-3 FAs	1 g Olive oil	3851	1 Year	100%	1.25 (0.90–1.72)		0.95 (0.56–1.60)		1.21 (0.96–1.52)	
Alpha Omega Trial [55]	226 mg EPA + 150 mg DHA	1.9 g ALA or placebo	4837	40 Months	100%	1.01 (0.82–1.24)	0.95 (0.68–1.32)			1.1 (0.87–1.17)	
GISSI-HF [56]	1 g/d n-3 FAs	Placebo	6975	3.9 Years	41.8%	0.91 (0.83–0.99)		0.93 (0.79–1.08)			
ORIGIN [64]	1 g n-3 FAs	1 g Olive oil	12536	6 Years	59%	0.98 (0.89–1.07)		1.10 (0.93–1.30)		1.01 (0.93–1.10)	0.92 (0.79–1.08)
OFAMI [74]	4 g n-3 FAs	4 g Corn oil	300	18 Months	100%	1.0 (0.45–2.2)		1.0 (0.39–2.6)	1.4 (0.7–2.6)		1.1 (0.84–1.3)
IEIS-4 [67]	1.08 g EPA	2.9 ALA or placebo	360	1 Year	100%		0.52 (0.29–0.95)	0.24 (0.05–1.1)	0.52 (0.3–0.9)	0.71 (0.48–1.1)	

RCTs, unblended											

DART [52]	Fish 200–400 g/week	Fruits and vegetables	2033 male	2 Years	100%	0.71 (0.54–0.93)				0.84 (0.66–1.07)	
GISSI [53]	1 g/d n-3 FAs	Vitamin E or placebo	11324	3.5 Years	100%	0.79 (0.66–0.93)	0.65 (0.51–0.82)	0.55 (0.51–0.82)	0.91 (0.68–0.94)	0.80 (0.68–0.94)	1.2 (0.81–1.9)
JELIS [61]	1800 mg EPA	Statin	18645	4.6 Years	20%	1.09 (0.92–1.28)	0.94 (0.57–1.56)	1.06 (0.55–2.07)	0.75 (0.54–1.04)	0.81 (0.69–0.95)	1.02 (0.91–1.13)
DART 2 [75]	2 meals of fish/week or 3 g n-3 FA suppl.	Intake of fruits, vegetables, and oats	3114	3–9 Years	100%	1.15 (0.96–1.36)	1.26 (1.0–1.56)	1.54 (1.06–2.23)			

Observational											

Nurses' Health Study [66]	Intake of fish (meals/week)		84688	16 Years	0%		0.55 (0.33–0.90)		0.73 (0.51–1.04)	0.66 (0.50–0.89)	
The Physicians' health study [71]	Intake of fish (meals/week)		21185	4 Years						1.2 (0.6–2.2)	
The Zutphen study [70]	Intake of fish (meals/week) or estimated EPA + DHA/d		1373	40 Years	0%		0.73 (0.47–1.13)	0.89 (0.34–2.30)			
JPHC study [60]	Quintiles of fish intake		41578	10 Years	0%		1.08 (0.42–2.76)	1.14 (0.36–3.63)	0.43 (0.23–0.81)		
WENBIT [63]	Quartiles of n-3 FA intake		2412	57 Months	90%	1.11 (0.73–1.67)	1.35 (0.75–2.42)				

**(b):** 

Study	Inclusion criteria	Prior MI/CAD	Dose of FAs	Control	*N*	Followup	Endpoint	Event rate (%)
RCTs, blinded								

Leaf et al. [47]	ICD due to SCA, spontaneous or inducible sustained VT.	78%	4 g/day n-3 FAs	4 g corn oil	402	12 months	Time to first ICD-event for VT/VF	Rate of ICD-event: 28% (n-3 FAs) versus 39%, RR 0.67 (0.47–0.95)
Brouwer et al. [78] (SOFA)	One episode of spontaneous VT or VF in the preceding year, ICD implanted	70%	0.9 g/day n-3 FAs	2 g high-oleic acid sun-lower oil	546	12 months	Appropriate ICD intervention for VT or VF, or all-cause death	30% versus 33% with sustained ICD intervention or death HR 0.86 (0.64–1.16)
Finzi et al. [79] (GISSI-HF)	ICD due to SCA, sustained VT or for primary prevention of syncope.	41.7%	1 g/day n-3 FAs	Placebo	566	928 days	Incidence of ICD-interventions	ICD events 27.3% versus 34.0%, HR 0.80 (0.59–1.09). Mortality 26.6% versus 24.3%, HR 1.25 (0.89–1.75)
Raitt et al. [80]	ICD and a recurrent episode of VT or VF.	73%	1.8 g/day n-3 FAs (1.3 g EPA/ DHA)	Olive oil (73% oleic acid)	200	2 years	Time to first ICD-event for VT/VF and frequency of recurrent VT/VF events	65% versus 59% with ICD therapy. Recurrent VT/VF more common in n-3 FA group (*P* < 0.001)

RCTs, non-blinded								

Madsen et al. [81]	Inducible sustained monomorphic VT	83%	3.9 g n-3 FAs	0.9% saline	6		Level of stimulation required to induce monomorphic VT	2 of 6 noninducible 2 of 6 increased stimulation required

Intervention studies, non-randomized								

Schrepf et al. [45]	Repeated episodes of sustained VT	90%	3.8 g n-3 FAs as IV infusion		10		Inducibilty of sustained VT in patients with a positive test at baseline	2 of 7 patients (29%)
Metcalf et al. [44]	ICD and inducible sustained monomorphic VT	100%	3 g/day n-3 FAs	No dietary manipulation	26	6 weeks	Level of stimulation required to induce monomorphic VT	42% versus 7% without inducible VT.

Observational								

Aarsetoey et al. [82]	SCA with documented VF during the ischemic phase of an MI	100%	Blood omega-3 index	Omega-3 index in MI patients without SCA	195		The omega-3 index in SCA patients versus MI patients free of SCA	1% increase of the omega-3 index associated with 48% reduction in risk of VF

RCT: randomized controlled trial, EPA: eicosapentaenoic acid, DHA: docosahexaenoic acid, ALA: alpha-linolenic acid, VT: ventricular tachycardia, VF: ventricular fibrillation, and SCA: sudden cardiac arrest.

**Table 3:** Official recommendations for the use of marine n-3 fatty acids in CVD prevention.

	Primary prevention	Secondary prevention
The American Heart Association (AHA)^1^	Eat a variety of fish, preferably oily fish (salmon, tuna, mackerel, herring, and trout), at least twice a week. Consuming fish oil supplements should only be considered by people with high levels of triglycerides who consult with their physicians.	Consume about 1 gram per day of the fish oils EPA and DHA (eicosapentaenoic and docosahexaenoic acids), preferably from oily fish, although EPA + DHA supplements could be considered in consultation with their physicians. People who have elevated triglycerides may need two to four grams of EPA and DHA per day provided as capsules under a physician's care.

European Society of Cardiology (ESC)	Eat fish twice a week, of which once oily fish^2^. The recommended doses of total EPA and DHA to lower triglycerides have varied between 2 and 4 g/day. Use of n-3 fatty acids (prescription products) as an adjunct to the diet if triglycerides exceed 5.6 mmol/L (496 mg/dL)^3^.	Eat fish twice a week, of which once oily fish^2^. The recommended doses of total EPA and DHA to lower triglycerides have varied between 2 and 4 g/day. Use of n-3 fatty acids (prescription products) as an adjunct to the diet if triglycerides exceed 5.6 mmol/L (496 mg/dL)^3^.

American College of Cardiology (ACC)	No recommendation.	Encourages increased consumption of omega-3 fatty acids in the form of fish or in capsule form (1 g/d) for risk reduction. For treatment of elevated triglycerides, higher doses are usually necessary for risk reduction^4^.

International Society for the Study of Fatty Acids and Lipids (ISSFAL)^5^	A minimum intake of eicosapentaenoic acid (EPA) and docosahexaenoic acid (DHA) combined, of 500 mg/d.	No recommendation.

Scientific Advisory Committee on Nutrition, United Kingdom (UK SACN)^6^	Recommends the equivalent of 450 mg marine omega-3 daily and an increase in population oily fish consumption to one portion a week.	Recommends the equivalent of 450 mg marine omega-3 daily and an increase in population oily fish consumption to one portion a week.

World Health Organization (WHO)^7^	Regular fish consumption (1-2 servings per week) is protective against coronary heart disease and ischaemic stroke and is recommended. The serving should provide an equivalent of 200–500 mg of eicosapentaenoic and docosahexaenoic acid.	Regular fish consumption (1-2 servings per week) is protective against coronary heart disease and ischaemic stroke and is recommended. The serving should provide an equivalent of 200–500 mg of eicosapentaenoic and docosahexaenoic acid.

^1^ http://www.heart.org/.

^2^European Guidelines on cardiovascular disease prevention in clincal practice (version 2012). The fifth joint task.

Force of the European Society of Cardiology and Other Societies on Cardiovascular Disease Prevention in Clinical Practice. EHJ 2012; 33: 1635-1701.

^3^ESC/EAS Guidelines for the management of dyslipidaemia. The Task Force for the management of dyslipidaemias of the European Society of Cardiology (ESC) and the European Atherosclerosis Society (EAS). EHJ 2011; 32(14): 1769-1818.

^4^AHA/ACC Guidelines for Secondary Prevention for Patients with Coronary and Other Atherosclerotic Vascular Disease: 2006 update endorsed by the National Heart, Lung, and Blood Institute. JACC 2006; 47(10): 2130-9.

^5^ http://www.issfal.org/.

^6^Advice on Fish Consumption: Benefits and Risks was published in 2004 by the joint SACN/COT Subgroup (SACN 2004).

^7^World Health Organization (WHO) 2003: “Diet, Nutrition and the Prevention of Chronic Diseases.”
